# Genetically modified large animal models for investigating neurodegenerative diseases

**DOI:** 10.1186/s13578-021-00729-8

**Published:** 2021-12-21

**Authors:** Weili Yang, Xiusheng Chen, Shihua Li, Xiao-Jiang Li

**Affiliations:** Guangdong Key Laboratory of Non-Human Primate Research, Guangdong-Hongkong-Macau Institute of CNS Regeneration, Jinan University, Guangzhou, 510632 China

**Keywords:** Animal models, Species, Neurodegeneration, Pathogenesis, CRISPR/Cas9

## Abstract

Neurodegenerative diseases represent a large group of neurological disorders including Alzheimer’s disease, amyotrophic lateral sclerosis, Parkinson’s disease, and Huntington’s disease. Although this group of diseases show heterogeneous clinical and pathological phenotypes, they share important pathological features characterized by the age-dependent and progressive degeneration of nerve cells that is caused by the accumulation of misfolded proteins. The association of genetic mutations with neurodegeneration diseases has enabled the establishment of various types of animal models that mimic genetic defects and have provided important insights into the pathogenesis. However, most of genetically modified rodent models lack the overt and selective neurodegeneration seen in the patient brains, making it difficult to use the small animal models to validate the effective treatment on neurodegeneration. Recent studies of pig and monkey models suggest that large animals can more faithfully recapitulate pathological features of neurodegenerative diseases. In this review, we discuss the important differences in animal models for modeling pathological features of neurodegenerative diseases, aiming to assist the use of animal models to better understand the pathogenesis and to develop effective therapeutic strategies.

## Introduction

Neurodegenerative diseases such as Alzheimer’s disease (AD), Parkinson’s disease (PD), Huntington's disease (HD), and Amyotrophic lateral sclerosis (ALS) are incurable and have been one of the most challenging health issues. The common feature of these diseases is a progressive loss of specific populations of neurons in the aging human brain [1]. With increasing life expectancies, the incidence of neurodegenerative diseases is rapidly growing worldwide.

AD, which is the most common form of neurodegenerative diseases, affects about 7–8% people over age 65. The main clinical manifestations of AD include progressive memory loss, cognitive dysfunction, behavioral disorders, and other related impairments. Neuropathologically, AD is characterized by extracellular senile amyloid plaques and intracellular neurofibrillary tangles (NFTs), along with other molecular changes such as neuroinflammation, brain atrophy, synaptic pathologies, and cerebral amyloid angiopathy [2, 3].More than 90% of patients with AD are sporadic cases and show dementia in their mid-60 s and later, and less than 10% of AD cases have the early-onset form of diseases that can be caused by a single genetic mutation in the APP genes (Presenilin 1, Presenilin 2, and Amyloid precursor protein APP) [4]. Parkinson’s disease (PD) is the second most common neurodegenerative disorder that affects more than 1% people over age 60. The pathologic hallmarks of PD are the preferential loss of dopamine (DA) neurons and formation of Lewy body inclusions in the substantia nigra pars compacta [5]. Like AD, the majority of PD cases are sporadic, and mutations in the genes encoding alpha-synuclein, PINK1, Parkin, LRRK2 and others have been found in 10–15% of familial PD cases [6]. ALS is also a progressive neurodegenerative disease that particularly affects motor neurons in the brain and the spinal cord, resulting in the loss of muscle movement [7]. Similar to AD and PD, most ALS patients are sporadic, and about 5%-10% of patients suffer from the familial form of ALS. The familial ALS could be caused by various mutations of genetic loci, including TAR DNA-binding protein 43 (TDP- 43), superoxide dismutase 1 (SOD1), fused in sarcoma (FUS), and C9ORF72 [8, 9]. On the other hand, HD shows autosomal dominance with full penetration, which is caused by a CAG repeat expansion (> 36 CAGs) in exon 1 of the HD gene that is translated to a polyglutamine (polyQ) repeat in the disease protein huntingtin (HTT) [10, 11].The polyQ expansion causes HTT to misfold and aggregate in the patient brain, resulting in the preferential loss of the medium spiny neurons in the striatum and extended neurodegeneration in various brain regions as HD progresses [11]. Currently, effective therapies are still lacking for these neurodegenerative diseases, and no proven treatment can halt or slow the progression of these diseases. Animal models that can recapitulate key pathological changes that occurring in the patient brains would be important for developing effective therapeutic strategies.

### The emerging need to use large animal models to study neurodegenerative diseases

The genetic mutations identified in the neurodegenerative disease genes make it possible to use genetic manipulation for modeling these diseases in animals, which are essential to our understanding of the disease pathogenesis. A variety of genetically modified animal models, mostly in rodents, have been generated to study the pathogenesis and therapeutics for neurodegenerative diseases. The rodent models have provided important insights into the pathogenesis of neurodegenerative diseases. For example, the mouse models can remarkably recapitulate protein misfolding and aggregation seen in the patient brains [12–14]. However, most of the mouse models cannot fully mimic the symptoms and pathologies of neurodegenerative diseases. For example, although the current HD mouse models show age-dependent accumulation of mutant HTT and associated neurological symptoms, they lack overt and selective medium spiny neuronal loss that is seen in HD patients [15, 16]. Similar to HD mouse models, the majority of AD transgenic mouse models have no overt neuronal loss in the cortex and hippocampus, and nearly all the PD transgenic mouse models have no obvious loss of dopamine (DA) neurons, a pathologic hallmark of PD [17, 18].

The differences in neuropathology between rodent models and patient brains with neurodegenerative diseases could be due to species differences determined by genomic, molecular, and anatomic differences between rodents and humans (Table 1). The brain development is considerably different in mouse and human: the human brain requires more than 10 months to fully develop, whereas the formation of mouse brain only takes 21 days [19]. As a result, the brain structures in large animals and rodents are also noticeably different. For example, the rodent brains lack gyrification that exists in the brains of large mammals (such as pig, monkey and human). Another example is that the striatum, which is the most affected region in HD, consists of the caudate nucleus and putamen in large animal brains while these two parts are indistinguishable in rodents [20]. The differences in anatomical structure and neuropathology between rodent and human brains highlight the demand for using larger animals that are closer to humans for modeling neurodegenerative diseases. Particularly, when CRISPR-Cas9 is available to edit the genes in large animals to mimic human genetic mutations, it becomes more feasible to generate large animal models of neurodegenerative diseases.

**Table 1:** Species-dependent differences

Species	Sexual maturity	Gestation period	Average life span (year)	Average weight (kg)
Human	15–18 years	266 days	75	50
Rhesus monkey	3–5 years	165 days	25	6
Pig	9–11 months	114 days	7	80
Mouse	6–8 weeks	19–21 days	2	0.03

### Genome editing in large animals

Although the modern transgenic methods have been used to establish large animal models, the transgene was randomly introduced into the chromosomes and may not yield the endogenous expression level of mutant genes. Furthermore, the following limitations make it difficult to create large animal models that can mimic germline transmissible mutations: the lack of embryonic stem cells (ESCs) for in vitro genome editing, the low efficiency of homologous recombination, and the long-life cycle. Thus, the recently developed nuclease-mediated genome editing technology (CRISPR/Cas9) that can modify the endogenous genome makes it feasible to expand genetic engineering to many species, especially large animals.

**Fig. 1:**
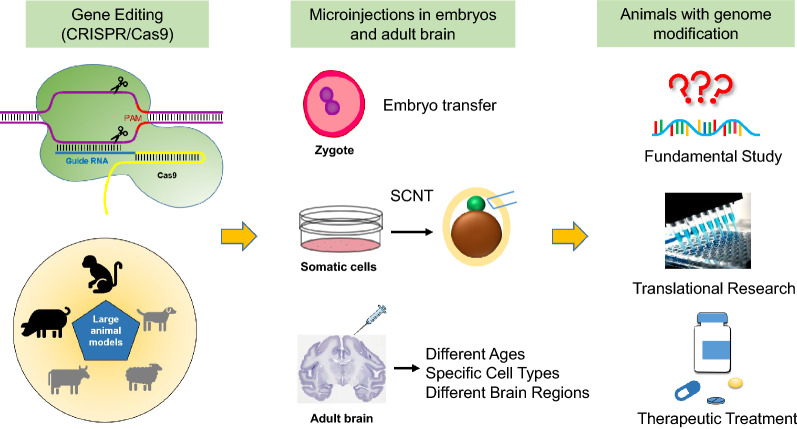
Genome editing in large animals. CRISPR/Cas9-mediated gene targeting can occur in zygotes, somatic cells, and adult brain cells, enabling generation of various animal models that carry different genetic mutations

CRISPR/Cas9 has now been used for genome editing in non-human primates [21–24]. The development of base editor system [25, 26] is particularly useful to introduce a point mutation in the endogenous genes [27, 28]. These new gene-editing tools can efficiently modify the endogenous genome of a variety of species in vitro and in vivo and can be used with embryonic stem cell culture, somatic nuclear transfer, and brain stereotaxic injection to edit genes in embryos and adult cells (Fig. 1). Thus, although embryonic stem cells of large animals are still not available for genome editing, CRISPR/Cas9 in combination with other technology has enabled the establishment of several large animal models that harbor genetic mutations found in humans.

**Fig. 2:**
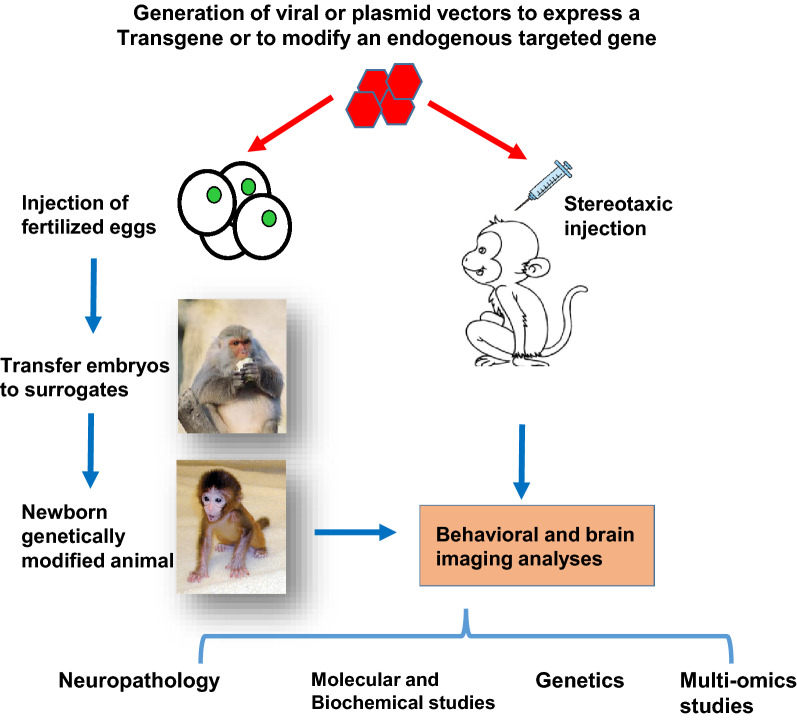
Strategies to generate monkey models of neurodegenerative diseases. Genetic modifications in monkey embryos can be achieved by microinjection of transgene or CRISPR/Cas9 to target the endogenous gene. Alternatively, stereotaxic injection of viral vectors expressing transgene or CRISPR/Cas9 into specific brain regions of adult monkey can result in brain region-dependent gene editing

For generating monkey models, germline genome editing can be achieved by injection of transgene or CRISPR/Cas9 into the fertilized eggs. However, the long-life cycle and the high costs of the non-human primates prevent the widespread use of this important animal model. Since CRISPR/Cas9 can also target genes in adult neuronal cells [29–31], it can be applied to the brains of adult monkeys via stereotaxic injection of viral expression vectors. Such studies would allow one to explore the function of mutant genes in adult monkey and also to more rapidly generate monkey models that can mimic brain region-specific neurodegeneration (Fig. 2).

Pig is another commonly used large animal that shares considerable similarities with humans in body size, organ(s) physiology and anatomical dimensions. In comparison to non-human primates, there are several advantages of pigs: generation of multiple piglets (6–12 piglets from a single sow), short gestation time (about 120 days), and relatively lower costs [32]. Importantly, pig models can be established using somatic cell nuclear transfer (SCNT) to introduce genetic mutations in the endogenous pig genes to generate knock-in and knock-out models [20, 33–35]. Thus, pigs have become important in modeling a number of human diseases and are also used in human organ xenotransplantation.

### Large animal models of Huntington's disease

Genetically modified large animal models of HD, PD, ALS, and AD are summarized in Table 2. Of these animal models, HD transgenic monkey model was the first transgenic monkey model of human diseases, which was established by injecting lentiviruses into fertilized rhesus monkey oocytes to express exon1 (1–67) mutant HTT containing 84Q [36]. Because of the monogenetic mutation (CAG repeat expansion) in HD, expressing expanded CAG repeats in animals has been used to generate a variety of HD animal models. Unlike transgenic HD mice that express the same transgenic HTT and can survive after birth without overt neurodegeneration [37], the HD transgenic monkeys died postnatally and showed severe neurodegeneration [36]. Transgenic HD pigs that express N-terminal mutant HTT (1–208) containing 105Q were then generated via somatic nuclear transfer, and most of the transgenic HD piglets also died postnatally and showed apoptotic cells in their brains [38]. However, the transgenic mice expressing the same mutant HTT fragment could live up to 9 months [38], suggesting the N-terminal mutant HTT is more toxic in larger animal models.

**Table 2:** Large animal models of neurodegenerative diseases

Disease	Genetic anomaly	Species	Modifications Approach	Pathology and phenotypes	References
Huntington’s Parkinson’s Alzheimer’s	HTT	Minipig	Lentiviral mediated Transgenesis of mHTT (N548)	- No aggregate formation and reduced intensity of DARPP32 immunoreactivity at age of 16 month - No developmental or gross motor deficits up to 40 months of age	[39]
	HTT	Minipig	CRISPR gene editing/SCNT: Knock-in HD-150Q	- Age-dependent neurological symptoms including body weight loss, early death, and movement difficulties - Selective neurodegeneration in the striatum	[20]
	HTT	Rhesus macaque	Lentiviral mediated transgenesis of mHTT (exon1 and N512)	- Postnatal death, key clinical HD features including dystonia, chorea, and seizure - Severe neurodegeneration	[36]
	HTT	Sheep	Embryo DNA microinjection/transgenesis	- Decreased expression of the neuronal marker DARPP-32 in medium-sized spiny neurons in the striatum at 7 months - Grow normally	[41]
	SNCA	Primate	Transgenic: overexpression	- Age-dependent non-motor symptoms (cognitive defects, anxiety phenotype and poor fine finger coordination)	[88]
	PINK1/Parkin/DJ-1	Pig	KO	- No obvious neuronal loss - Normal behavior	[53]
	PINK1/Parkin	Pig	KO	- No obvious neuronal loss - Normal behavior	[35]
	PINK1	Rhesus monkeys	KO	- Severe neuronal loss - Reduced motor activity	[24]
	PINK1/DJ-1	Rhesus monkeys	KO (adult)	- Classic PD symptoms, - Severe nigral dopaminergic neuron loss - α-synuclein pathology	[55]
	PINK1	Rhesus monkeys and Cynomolgus monkeys	KO (adult)	- Severe neuronal loss - Motor deficits	[31]
	APP	Pig	Transgenic	- No pathological changes over 5 years	[81, 82]
	PSEN1	Pig	Transgenic	- No AD-like pathological changes over 3 years	[83]
	APP	Cynomolgus monkeys	Transgenic	- Model established and ongoing observation	[89]
ALS	hSOD1	Pig	Transgenic (SCNT)	- No ALS-like phenotype was reported - Normal development of founder pigs	[90]
	hSOD1	Pig	Transgenic (SCNT), CMV promoter	- Movement deficits, limb muscle atrophy - Loss of motor neurons from age 8 months - Formation of neuronal intranuclear inclusions	[73]
	TDP-43	Pig	Transgenic	- Severe phenotypes and early death	[67]
	TDP-43	Rhesus monkeys	Overexpression in adult brain	- Cytoplasmic accumulation of mutant TDP-43 - Paralyzed limb	[68]

Transgenic pigs expressing a large fragment of mutant HTT (1–548) containing 124Q were also generated by lentiviral infection of porcine embryos. However, these transgenic HD pigs showed much milder phenotypes and did not die after birth [39, 40]. Because transgene expression is largely controlled by the promoter that drives transgene expression and copy numbers as well as chromosomal location of the transgene, it is possible that the transgenic HTT expression level in these HD pigs is not high enough to induce early and severe neuropathology. In line with this possibility, transgenic sheep that express full-length mutant HTT with 73Q and were generated via microinjection into pronuclei of single-celled zygotes show very mild phenotypes [41]. Because somatic nuclear transfer ensures the transgene expression in each cell while viral infection or microinjection of fertilized oocytes can lead to various degrees of transgene expression in different types of cells, the different phenotypes in transgenic HD animals are clearly dependent on the transgene expression.

To overcome the limitation of transgenic approach, a HD knock-in pig model was generated via CRISPR/Cas9 and somatic nuclear transfer, which expresses an expanded polyCAG (150 CAG) in the pig HTT gene and precisely mimics the genetic mutation that occurs in the endogenous HTT gene [20]. Importantly, when full-length mutant HTT with 150Q is endogenously expressed in this HD pig model, it causes striking and selective neurodegeneration as well as movement disorders, effectively recapitulating the typical pathological and clinic features in HD patients. Furthermore, the expanded CAG repeats and neurological phenotypes of these HD KI pigs can be transmitted to next generations by germline [20], providing a valuable model for investigating the pathogenesis and therapeutics for HD.

### Large animal models of Parkinson's disease

Animal models developed to investigate the pathogenesis of PD fall into two categories: neurotoxic and genetic models. The neurotoxic models are mainly induced by the neurotoxins 6-hydroxydopamine (6-OHDA) (a hydroxylated analogue of dopamine) or 1-methyl-4-phenyl-1,2,3,6-tetrahydropyridine (MPTP), which cause dopamine neuronal loss in the substantia nigra (SN). The neurotoxic PD models are valuable for studying the pathogenesis of PD-associated neurodegeneration [42], but these models appear to have unstable phenotypes and cannot mimic the progressive process of neuronal loss and other pathologies in PD patients.

Although transgenic mouse and monkey models that overexpress mutant alpha-synuclein have been generated for investigating PD pathology and validated the neurotoxicity of mutant proteins [43–46], the various expression levels of exogenous mutant proteins could compromise the phenotype outcomes such that it is difficult to compare the merits of each transgenic PD animal model. Based on the fact that some genetic mutations also caused PD, a variety of mouse models that carry these genetic mutations in the endogenous genome were generated. However, none of them show the typical degeneration of dopaminergic neurons seen in the brains of PD patients [47–50]. For example, mutations of the PINK1 gene can result in loss of function and cause early-onset Parkinson’s disease (PD) with selective neurodegeneration [51]. Unfortunately, current PINK1 knockout (KO) mouse cannot yield the selective and overt neurodegeneration [48–50]. Similarly, deletion of the gene for Parkin, which works together with Pink1 in protecting against mitochondrial damage, did not produce any obvious degeneration either in the mouse brain [47, 50, 52].

Interestingly, disrupting the PINK1 and Parkin genes in pigs via CRISPR/Cas9 targeting did not produce any neurodegeneration and severe phenotypes either [35, 53]. However, CRISPR/Cas9 targeting the PINK1 gene in the monkeys resulted in phenotypic animals, though the phenotypes are dependent on the types of PINK1 mutations created. Chen et al. utilized the paired single guide RNA (sgRNA)/Cas9-D10A nickases to disrupt the monkey PINK1 in the fertilized monkey oocytes and found that targeting PINK1 exon 2 alone is not sufficient to model PD phenotypes in the live monkeys [54]. On the other hand, direct injection of AAV into the monkey substantia nigra to co-edit the PINK1 exon 3 and DJ1 genes could result in severe nigra dopaminergic cell loss and motor function deficits [55]. To ensure that PINK1 expression and function are completely lost, we used two gRNAs to disrupt the monkey PINK1 exon 2 and exon 4, resulting in a large PINK1 DNA fragment deletion in the monkey embryos. The newborn monkeys showed severe neurodegeneration or died postnatally [24], demonstrating for the first time that PINK1 is essential for neuronal survive in the primate brain. Further investigation identified that PINK1 functions as a cytoplasmic kinase, rather than a mitochondrial protein for mitophagy, to maintain the neuronal survival in the non-human primate [31]. Thus, investigation of the non-human primate model has uncovered important function of PINK1 and the associated pathological changes due to loss of PINK1, which cannot be identified in the mouse models.

### Large animal models of ALS

Because mutations in the nuclear TAR DNA-binding protein 43 (TDP-43) gene cause ALS, animal models carrying TDP-43 mutations were generated to investigate ALS pathogenesis. TDP-43 is a nuclear protein that is involved in a variety of cellular functions including gene transcription, RNA processing, and protein homeostasis [56–58]. In patient brains with ALS, fronto-temporal lobar degeneration (FTLD), and other neurological disorders, TDP-43 is accumulated and forms aggregates in the cytoplasm [8, 58, 59]. This cytoplasmic redistribution of TDP-43 in human brains can lead to a loss of function in nucleus and a gain of toxicity in cytoplasm [60, 61]. However, in the brains of most transgenic mice, TDP-43 is still predominantly localized in the nucleus [60–64]. Although some mouse models can show the minimal level of cytoplasmic TDP-43 [63, 65, 66], this minimal distribution of cytoplasmic TDP-43 does not mimic the major pathological hallmark of cytoplasmic mislocalization of TDP-43.

Our group has previously created the TDP-43 transgenic pig model that displays the cytoplasmic distribution of TDP-43 [67]. We also used the non-human primate to investigate the subcellular distribution of mutant TDP-43 via directly delivering viral vector expressing mutant TDP-43 into the rhesus monkey brain cortex and substantia nigra [68]. For comparison, we injected the same viral vector into the mouse brain. Comparison of the subcellular localization of mutant TDP-43 in the brains of mice and rhesus monkeys revealed that the majority of mutant TDP-43 remained in the nuclei of the mouse brain but was mainly distributed in the cytoplasm of the monkey brain [68]. The cytoplasmic distribution of mutant TDP-43 in the monkey brain is consistent with the previous finding that transgenic TDP-43 is distributed in the neuronal cytoplasm in the monkey spinal cord [69]. Furthermore, the primate-specific caspase-4 was found to cleave TDP-43 in the monkey brain to mediate the cytoplasmic accumulation of TDP-43 by removing its NLS-containing N-terminal domain [68]. Thus, the findings in large animal models indicate that differential subcellular localization of mutant TDP-43 is species-dependent rather than brain region-dependent.

The human copper/zinc superoxide dismutase 1 (SOD1) mutations also cause familial ALS. However, transgenic mutant SOD1 mouse models do not show the intranuclear inclusions seen in the brains of ALS patient with SOD1 mutations [70–72]. To investigate the effect of mutant SOD1 in large animals, Yang et. al generated the SOD1 (G93A) transgenic pigs via the SCNT method [73]. The transgenic SOD1 pigs show motor function defects and neuronal degeneration. More importantly, mutant SOD1 was accumulated in the nucleus to form ubiquitinated nuclear aggregates at the early disease stage [73]. The differences between transgenic SOD1 mice and pigs further support the idea that large animal models can more faithfully mimic the pathological changes seen in the patient brains.

### Large animal models of Alzheimer's disease

According to etiology, most AD patients are sporadic, and the familial type of AD only accounts for 5–10% of the total AD cases. The familial type of AD is mostly correlated with the genetic mutations in the genes for amyloid precursor protein (APP), Presenilin-1 (PSEN1), Presenilin-2 (PSEN2) [4]. Until now, the exact mechanism of AD remains elusive, largely due to the lack of animal models that can adequately mimic the AD pathology in humans. Various species of animal models for AD have been investigated, including fruit flies [74], rodents [75], dogs [76], pigs and non-human primates [77]. Among those species, the most widely used AD models are from rodents. However, the pharmaceutical treatments that were developed and effective in rodents have consistently failed to show obvious effects on humans in clinical trials. It is also noteworthy that rodents do not express the isoforms of Tau that leads to intracellular neurofibrillary tangles (NFTs), a hallmark of AD, such that multiple transgenes were required for co-expression to model AD pathologies in rodents [78–80]. The mini pigs expressing APP or PSEN1 engineered by nuclear transfer technology have failed to show any pathological changes at 2 and 3 years of age [81–83]. Because aging is one of the biggest risk factors for the development of AD, the lack of pathology and behavioral changes of AD pig models may be due to the early time points selected for analysis.

Because the non-human primates are much closer to humans, they should be the most biologically relevant model to study Alzheimer’s disease. Interestingly, no case of AD in naturally long-lived non-human primates has been reported [84]. The older NHPs could develop natural pathogenesis with senile plaques and Tau protein aggregations, mild cognitive deficits, but do not show overt and widespread neuronal loss as seen in AD patients [84, 85]. Microinjection of soluble and fibrous amyloid beta peptide (Aβ) has also been used to induce AD in non-human primate, revealing that the neurotoxicity of Aβ injection is dose-dependent and age-related because young monkeys did not develop visible changes of neuronal bodies or axons [86, 87].

Since ageing is a crucial pathogenic factor in these neurodegeneration diseases, the disease phenotypes might take longer time to appear in large animal models. Species-specific factors are also likely to influence the disease progression. These possibilities should be explored by establishing more large animal models, especially the non-human primate models, that carry the AD genes and are able to show the AD phenotypes.

### New insight from large animal models

Animal models are an important tool for studying pathogenesis, disease progression, and therapeutic treatments. A successfully established animal model of neurodegenerative disease should adequately recapitulate the clinical characteristics and pathology of patients. However, such an animal model is still lacking for most neurodegenerative disease. Rodents are the most widely used animal model for generating disease models owing to their relatively short life span, easier and rapid breeding, and lower costs. However, rodents have only 48–66% genetic homology with humans and show considerable differences to humans in brain size and development [19, 91]. The current large animal models reviewed above have demonstrated important differences in neuropathology when compared with the rodent models. The striking neurodegeneration phenotypes seen in HD KI pigs and PINK1 mutant monkeys highlight the species-dependent influences on the development of neurodegeneration. Such influences could be due to species-specific expression of the disease genes or modifiers. Comparison of PINK1 KO models of mouse, pig and monkey also underscores the unique function of PINK1 in the primate brains because PINK1 is undetectable in the mouse brain but abundantly expressed in the primate brain at the protein level, which may explain why Pink1 knock out mouse models do not have overt neurodegeneration [31]. We also found that PINK1 is expressed as a kinase form that is vital to neuronal survival in the primate brains [24, 31]. Thus, genetic mutations such as a point mutation or a mutation in the non-kinase domain of PINK1 created in other monkey models may not be able to significantly affect PINK1 kinase function to elicit obvious neurodegeneration [54].

In addition to gene editing that can induce embryonic mutations in large animals by embryo microinjection, stereotaxic injection of viral expression vectors can be applied to modify genes in specific brain regions in adult animals at different ages to assess brain regional effects or age-dependent effects [31, 43, 55]. Such studies would be particularly useful for large animals to mimic distinct and age-dependent neuronal loss in neurodegenerative diseases. Also, editing genes in different cell types (such as neurons or glia) by using specific promoters to express CRISPR/Cas9 would allow us to investigate gene functions in different types of brain cells. The findings in large animal models are expected to offer new insights into pathogenesis and therapeutics of neurodegenerative diseases.

## Limitations and challenges of using large animal models

The application of gene editing technology, especially the CRISPR/Cas9 system, makes revolutionary changes in modifying genomes in large animals for investigating neurodegenerative diseases. However, there are still many challenges to overcome. One potential challenge of CRISPR/Cas9 system is the mosaic mutations, which may result from various types of mutations created by the Cas9 nuclease. The mosaicism in offspring can be reduced by outcrossing the mosaic founders with wild type animals. However, it will take years to eliminate mosaicism in large animal models, especially for the non-human primates that require 4–5 years of sexual maturity to produce next generation. Thus, the mosaic targeting issue should be considered when evaluating the phenotypes of large animal models, because the funder animals that carry CRISPR/Cas9-mediated mosaic mutations are often used for investigation. The mosaic issue could potentially be improved by shortening the half-life of Cas9 expression after cell division such as tagging Cas9 with ubiquitin proteasomal degradation signals [92]. Other possible strategies using a combined strategy in early stage zygotes or germline cells to reduce mosaicism have also been discussed in a recent review [93]. For example, the gene editing tools combined with somatic cell nuclear transfer have been successfully used in establishing the pig models that carry the same single genetic mutation as in human patients. Liu et al. has created two cynomolgus monkeys by somatic cell nuclear transfer (SCNT) [94], offering great promise for generating more gene targeting non-human primate models in the future.

Although the mosaicism often exists in the non-human primate models, it depletes gene expression to various extents, leading to various degrees of cellular phenotypes that are correlated with the extent of genetic elimination. Thus, using imaging and RNAseq analysis at the single cell resolution, one can take the advantage of mosaicism to analyze the correlation of genetic mutation and cellular phenotypes in the large animal models.

Possible off-target effects of CRISPR/Cas9 have been considered as another important issue because CRISPR/Cas9 relies on approximately 23 base pair matches [95]. Studies have reported that Cas9 could tolerate mismatches that are associated with their distribution and number [96–98]. Thus, designing specific sgRNAs and controlling Cas9 expression should minimize the off-targets and increase the specificity of CRSIPR/Cas9-mediated gene targeting.

An obvious concern for generating large animal models is the high cost and time consumed for such research. For example, pigs have a sexual maturity of 5–8 months and the gestation period of about 114 d. For monkeys, the sexual maturity takes 4–5 years and gestation period is about 165 d with normally delivering only one offspring per year. Alternatively, the direct administration of gRNA/Cas9 into specific brain regions in adult large animals could more rapidly recapitulate brain regional neuropathology. The important information gained from large animal models would be highly valuable for generating more humanized mouse models. For example, once we know the primate-specific factors that critically contribute to neurodegeneration, we can introduce these factors to the rodents to make rodent models to be able to display important pathological features. Such mouse models can be used to rigorously test the effects of therapeutic strategies on neurodegeneration.

## Conclusions

The newly developed genome editing tool has greatly advanced the progress of generating valuable large animal models for neurodegenerative disease research. These large animal models allowed one to discover important pathological events that otherwise do not occur in small animals. However, the generation and investigation of genetically modified large animal models are still challenging, largely due to the high cost of animals and time consumed experiments. We anticipate that further optimization of the existing genome editing system or the generation of new tools will improve the efficiency and accuracy of genome modification of large animals. Moreover, important insights from large animal models would help establish small animal models that can more faithfully recapitulate important pathological features for investigating pathogenesis and developing effective therapies.

## Data Availability

Not applicable.
